# Comparison of intra-articular lumbar facet joint pulsed radiofrequency and intra-articular lumbar facet joint corticosteroid injection for management of lumbar facet joint pain: A randomized controlled trial: Erratum

**DOI:** 10.1097/MD.0000000000007232

**Published:** 2017-06-08

In the article, “Comparison of intra-articular lumbar facet joint pulsed radiofrequency and intra-articular lumbar facet joint corticosteroid injection for management of lumbar facet joint pain: A randomized controlled trial”,^[1]^ which appeared in Volume 96, Issue 13 of *Medicine*, Drs Yun Woo Cho and Min Cheol Chang should have been affiliated with the “Department of Physical Medicine and Rehabilitation, College of Medicine, Yeungnam University, Daegu, Republic of Korea.”
